# Establishing a set of acceptable demographic questions for use in health research through public consultation

**DOI:** 10.1186/s40900-026-00836-1

**Published:** 2026-01-28

**Authors:** Emma Lidington, Morgaine Stiles, Jessica Maudsley, Jennifer Ching, Andy Deutsch, Natasha Lipman, Emily Williamson, Georgiana Synesi, Karen Poole, Eleanor Knuckey, Tahera Hussain, Christina Derksen, Hannah Drysdale, Laura White, Georgia Mannion-Krase, Cherrelle Salmon, Jane Rigney, Beth Stuart, Rebecca Lewis

**Affiliations:** 1 CRUK Cancer Prevention Trials Unit, Centre for Cancer Screening Prevention and Early Diagnosis, Wolfson Institute of Population Health, Queen Mary University of London, London, UK; 2 Clinical Trials and Statistics Unit at the Institute of Cancer Research, London, UK; 3 Independent Patient Representative, London, United Kingdom; 4 Centre for Evaluation and Methods, Wolfson Institute of Population Health, Queen Mary University of London, London, UK; 5 Centre for Cancer Screening Prevention and Early Diagnosis, Wolfson Institute of Population Health, Queen Mary University of London, London, UK; 6 Barts Cardiovascular Clinical Trials Unit (CVCTU), William Harvey Research Institute, Centre for Cardiovascular Medicine and Devices, Queen Mary University of London, London, UK

**Keywords:** Demographics, Inclusivity, Diversity, Patient and public involvement, Public consultation, Trial participation

## Abstract

**Background:**

The importance of inclusivity in health care and health research is increasingly recognised in the UK. However, there are currently no UK standards for collecting self-reported demographic data from research participants. To address this gap, we undertook a public involvement activity. We worked with patient and public involvement partners and members of the public to establish an acceptable set of demographic questions for adult participants, taken from national survey questions to ensure comparable data.

**Methods:**

Our project team, which included two patient and public involvement partners, selected demographic questions that covered characteristics protected by the UKs Equality Act 2010 or groups identified as potentially underserved in research. These questions covered health, disability, and unpaid care; education and employment; sexual orientation and gender identity; and ethnicity, language, and religion. We conducted four discussion groups to review the proposed questions with diverse members of the public. We explored their views on questions, the explanatory text for the purpose of data collection, data storage (i.e. pseudonymised or anonymous), the length of the question set and any missing topics.

**Results:**

Twenty-nine public contributors took part. Of these, at least ten were from a minority ethnic background and eleven had one or more disabilities or long-term health conditions. Five contributors were people of faith, three were members of the LGBTQIA+ community, and seven had experience of providing unpaid care. Of the 18 questions, three were removed and ten were modified. This resulted in a revised question set of 15 items.

**Conclusions:**

The implementation of this question set will help to standardise data collection across studies, increasing comparability and researchers’ ability to evaluate inclusivity. The demographic question set is now available to non-commercial researchers across the UK as part of a pilot study to evaluate and improve its utility and performance.

**Supplementary Information:**

The online version contains supplementary material available at 10.1186/s40900-026-00836-1.

## Abstract (plain language summary)

Demographic characteristics refer to information that describes a person, such as age, gender, ethnicity, and education. Collecting demographic characteristics in research helps us understand who is taking part, and who is not. This is needed to ensure equal access to research and to make sure that results are relevant to people from all backgrounds.

There are no standard questions used in UK research to ask people about their demographic characteristics. This is challenging because these questions can be sensitive and hard to answer. We worked with patients and members of the public to create a set of acceptable questions, making sure we ask the right questions in the right way.

We looked for existing national survey questions so that researchers can compare people taking part in their research with the UK population. We then met with patient and public involvement partners and members of the public to look at the questions we had found and agree which to use. We also looked at how questions were worded to make sure they were easy to understand. We changed the questions if needed based on their feedback.

Two patient and public involvement advisors were part of our project team and twenty-nine people from a range of backgrounds joined the discussion groups. The final question set has 15 questions. Ten questions were changed based on discussion group feedback. The question set is now being used by UK researchers in their studies. In the future, these researchers will share information with us about how they used the questions and how people completed them. This will help us check whether the questions work in different types of medical research and make improvements to produce a standard set of questions for future use.

**Supplementary Information:**

The online version contains supplementary material available at 10.1186/s40900-026-00836-1.

## Background

The importance of inclusivity in health care and health research is increasingly recognised in the UK, brought into sharp focus by disparities in vaccine uptake and outcomes across different communities during the COVID-19 pandemic [1].

One contributing factor to these disparities may be differences in trial participation amongst the UK population. Clinical trials should include participants representative of those affected by the condition being studied to ensure equitable access to new treatments and applicability of trial results to the whole population affected [2]. Lack of representation also widens health disparities and may undermine trust in research and health care [3]. The NIHR-INCLUDE project (National Institute of Health Research Innovations in Clinical Trial Design and Delivery for the Under-served) identified several groups who may be underserved by research through expert consensus, including patients and members of the public (Table 1) [4]. However, quantitative data to demonstrate underrepresentation of these groups in UK research are lacking.

**Table 1:** Equality Act 2010 protected characteristics and INCLUDE examples of underserved groups

Equality Act 2010 Protected Characteristics	INCLUDE Examples of Underserved Groups
• Age • Disability • Gender reassignment • Marriage & civil partnership • Pregnancy & maternity • Race, including colour, nationality & ethnic or national origin • Religion or belief, including lack of belief • Sex • Sexual orientation	*Groups by Demographic Factors (Age, Sex, Ethnicity, Education):* • Age extremes (e.g. under 18 and over 75) • Women of childbearing age • Different ethnic minority groups • Male/female sex (depending on trial context) • LGBTQ+/sexual orientation • Educational disadvantage *Groups by Social and Economic Factors:* • People in full-time employment • Socio-economically disadvantaged/unemployed/low income • Military veterans • People in alternative residential circumstances (e.g., migrants, asylum seekers, care homes, prison populations, traveller communities, the homeless and those of no fixed abode) • People living in remote areas • Religious minorities • Carers • Language barriers • Digital exclusion/disadvantage • People who do not attend regular medical appointments • People in multiple excluded categories • Socially marginalised people • Stigmatised populations • Looked-after children *Groups by Health Status:* • Mental health conditions • People who lack capacity to consent for themselves • Cognitive impairment • Learning disability • People with addictions • Pregnant women • People with multiple health conditions • Physical disabilities • Visually/hearing impaired • Too severely ill • Smokers • Obesity *Groups by Disease Specific Factors:* • Rare diseases and genetic disease sub-types • People in cancer trials with brain metastases

Recent work examining differences in representation by age, sex and ethnicity in UK randomised controlled trials found only 60% of studies reported ethnicity [5]. Interestingly, among the studies reporting this data, the research samples broadly represented the wider UK ethnic makeup, but older adults tended to be less well represented [5]. This differs from findings of ethnic minority underrepresentation in the United States and highlights the need to collect and report relevant demographic data across a range of characteristics [6–8].

Extensive work is ongoing to establish better standards for collecting ethnicity data [9]. However, to our knowledge, there is little work being done to address other characteristics beyond ethnicity which are likely to be even less frequently collected. For example, a recent review of 407 NIHR trial reports found that no studies collected disability as a demographic characteristic [10].

In the past, researchers have argued that data collection should be minimised to the information used in the main outcome analysis [11]. However, given the growing importance of inclusivity, the recognition of the complexities of intersectionality and the increasing demand for large and comprehensive datasets, there is a potential need to reconsider this mindset to ensure that sufficient information is collected to allow comprehensive assessment of impact across different groups, especially in the meta-analysis setting. Several journals, including the Lancet, JAMA and Trials, are now mandating reporting of demographic characteristics of trial participants as part of baseline data [12–14]. This highlights the need for increased transparency to ensure that trials and research project populations are representative of the disease(s) being studied.

Some demographic data can be collected from participants’ medical records, however, sex and gender can be conflated in NHS records. Other characteristics, like ethnicity, are often poorly recorded, not well correlated with self-reported data, and are disproportionately incorrect among UK minority groups [15–17]. Whilst there are ongoing efforts to improve routine recording of this data in the NHS [9], the known inaccuracies emphasise the importance of collecting self-reported data directly from research participants.

To our knowledge, there are no UK standards for collecting self-reported demographic data from research participants. Related question sets in the UK are generally designed to collect data from staff or research funding applicants, rather than research participants [18–20]. Some overseas organisations have implemented standards for use in research [21, 22], but questions are often country-specific due to differences in population demographics, health care, education, societal arrangements and historical contexts.

Enhanced demographic data collection would permit better reporting and evaluation of inclusivity against population-level data (or condition-specific data where available) upon trial completion. Furthermore, it could allow study teams to actively monitor diversity during recruitment to intervene early and potentially enhance inclusion from less well represented groups. Collection of this data would also enable researchers to better evaluate the effectiveness of strategies to improve inclusivity.

**Fig. 1:**
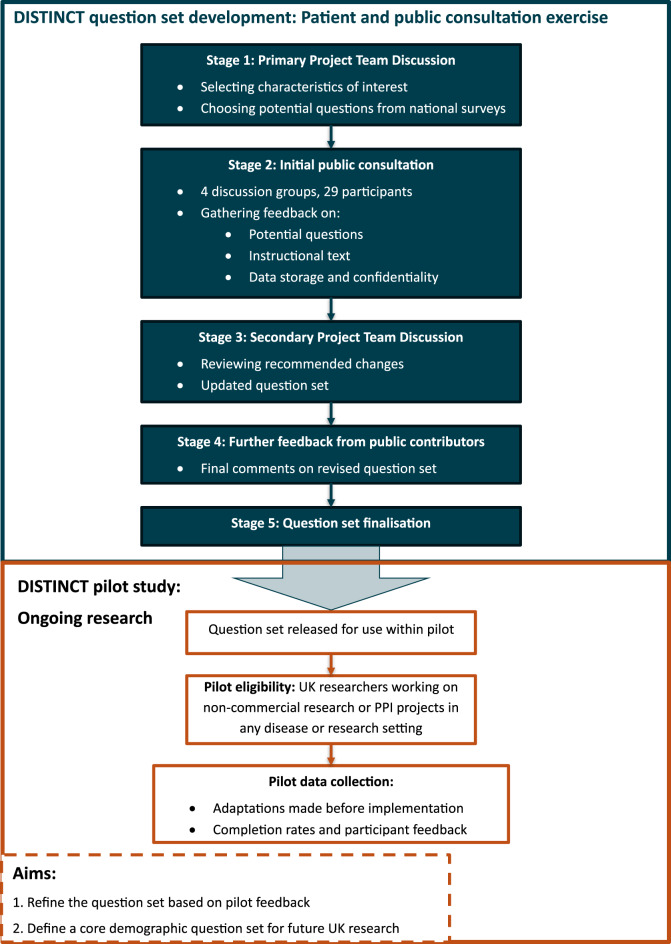
Overview of the DISTINCT project

To address these gaps, the DISTINCT project aimed to create a demographic question set for use in research through patient and public involvement (PPI) (Fig. 1). The project is co-led by the Cancer Prevention Trials Unit (CPTU) at Queen Mary University of London (QMUL) and The Clinical Trials and Statistics Unit at The Institute of Cancer Research (ICR-CTSU). The CPTU at QMUL conducts large scale screening, prevention and early diagnosis trials enrolling members of the general public, whilst the ICR-CTSU conducts treatment trials enrolling patients with cancer. Recognising the time and resource it takes to develop, test and validate questions, but equally the need to collect data more widely, we planned to use demographic items from existing national surveys and guidance where possible.

## Methods

There were five key stages involved in creating the DISTINCT demographic question set.

## Stage 1 – Primary project team discussion

### Selection of demographic characteristics

Potential demographic characteristics were carefully selected through a series of discussions within the project team, including triallists, social scientists, statisticians and patient and public involvement partners, over a six-month period. We considered the UK’s Equality Act 2010, which covers nine protected characteristics, and the INCLUDE guidance, which gives 35 examples of potentially underserved groups in health research (Table 1) [4, 23].

A total of 15 people were involved in these discussions. No formal consensus methods were used and EL and RL made the final decisions about characteristic selection.

### Item selection

Once the characteristics were agreed, we reviewed established national surveys to identify appropriate questions for each item. We considered whether UK population data was available for reference, whether the questions were used in other health research, the wording of the questions and response options, and the length and complexity of the questions. We prioritised questions with the most available evidence and comparable data, while limiting responder burden and maximising relevance to health research.

Where questions were available with extensive data which were considered appropriate for use in research, we used the questions exactly as they appeared in existing surveys. Where slight changes are known to be preferred (e.g., using ‘prefer to self-describe’ instead of ‘other’), these modifications were made. Where questions were available but measured a particular characteristic in more detail than might be needed for the purpose of monitoring inclusivity in research, the questions were simplified or combined. Where questions were unavailable, these were drafted by the project team (Table 3).

## Stage 2 – Initial public consultation

### Design

We undertook a consultation which included patient and public involvement partners and members of the public from diverse backgrounds to explore their views on the collection of demographic data in health research and the proposed question set. Although this was a public consultation rather than research, we used rapid qualitative techniques, which allowed us to systematically collate views throughout a series of discussion groups.

We aimed to hold at least four groups, each covering a different set of characteristics:

o Sexual orientation and gender identity
o Ethnicity, language, and religion
o Education and employment
o Health, disability, and unpaid care

Specific topics included:

o Overall acceptability of collecting this type of data
o How to describe the purpose of data collection
o Acceptability of the proposed questions and formats
o Views on storing the data anonymously versus linked to identifiable information
o Appropriate length of the question set
o Missing characteristics

Throughout the discussion groups, we also developed introductory text explaining the reason for data collection in an iterative process.

### Contributor identification

We created a flyer and invited potential public contributors through local and national patient and public involvement networks, cancer support charities, and advocacy groups (Appendix 1). Interested public contributors were asked to indicate which discussion group they were interested in attending and why. Contributors were only selected to attend one discussion group each, based on their interests, their description of themselves and their availability. Contributors were not asked to complete a demographic questionnaire as the development of an acceptable set of demographic questions was the purpose of the groups.

### Procedures

We planned to hold two-hour discussion groups online or in-person with eight to ten people per group. Discussion groups were led by EL and co-facilitated by three other team members including at least one of our patient and public involvement partners (MS, JM, EW, GS, CD, HD, CS, NL, AD).

Discussions were recorded for reference. All facilitators kept notes using a Rapid Assessment Process (RAP) sheet with sections for each item in the question set, views on the storage of data and the explanatory wording (Appendix 2). After each group, the facilitators met to discuss the key points, topics to discuss further in the next group and ways to improve the discussion. RAP sheets were then combined into a master sheet to track key feedback and address gaps in discussion.

At the end of the discussion groups, feedback from the master RAP sheet was summarised in a table alongside each question.

## Stage 3 – Secondary project team discussion

After the initial consultation feedback had been summarised, the project team discussed the recommendations and decided whether to make changes to the demographic questions and instructional text. EL and RL made the final decision on the questions to include and modify.

## Stage 4 – Requesting further feedback from our public contributors

After the question set had been revised, it was sent back to the public contributors by email for an additional review. They were asked to provide any further comments or suggestions by email. Simultaneously, contributors were sent a feedback form on their experiences in the discussion groups.

## Stage 5 – Question set finalisation

Once additional feedback had been received from public contributors, the project team discussed any proposed amendments in meeting and changes were agreed. The final question set was determined by EL and RL. Once the final version of the question set had been produced, it was then distributed to all collaborators and public contributors for their information.

## Ethical considerations and funding

This project was a patient and public consultation which did not require ethical approval or informed consent under UK regulations. Public contributor time was supported mainly by the Queen Mary University of London Centre for Public Engagement, with part funding from each trials unit involved.

## Results

### Selection of demographic characteristics

During stage 1, we decided to include all nine protected characteristics. The INCLUDE List was narrowed by considering groups which are most likely to be measurable, underserved in research and who might benefit most from participating in research (e.g., lack of access, worst outcomes). This final list is detailed in Table 2.

**Table 2:** Characteristics included in the question set reviewed in the public consultation

Protected Characteristics	INCLUDE Underserved Groups
• Age • Disability • Gender reassignment • Marriage & civil partnership • Pregnancy & maternity • Race, including colour, nationality & ethnic or national origin • Religion or belief, including lack of belief • Sex • Sexual orientation	• Educational disadvantage • People in full-time employment • Socio-economically disadvantaged/unemployed/low income • Carers • Language barriers • Learning disability • People with addictions (alcohol) • Physical disabilities • Visually/hearing impaired • Smokers

## Item selection

To identify potential questions, we reviewed an extensive list of surveys and guidance, including the Family Resources Survey, the 2021 England and Wales Census, Scotland’s 2022 Census, Office for National Statistics guidance, the National Statistics Socio-Economic Classification (NS-SEC), NIHR Diversity Data questions, Stonewall and LGBT Foundation guidance, the Diversity and Inclusion Survey (DAISY), and Race Equality Foundation recommendations [19, 20, 24–29].

We identified 18 commonly used questions in national surveys which we presented for public consultation (Table 3). After reviewing the questions available for alcohol use and smoking we decided the questions would likely only be relevant in specific disease settings, so we excluded these questions to limit the length of the question set.

**Table 3:** Evolution of the final question set

Selected question source and wording	Changes made to source question based on project team discussion	Revised question for public consultation	Final DISTINCT question following public consultation
Daisy questionnaire V2 **What is your age?** • Up to and including 24 years • 25–34 years • 35–44 years • 45–54 years • 55–64 years • 65–74 years • 75+ years • Prefer not to say	• Bespoke question created. • Reduced groups to 5-year categories. • Started at 16 as we were focusing on research in adults.	**Age** 16–19 55–59 20–24 65–69 25–29 70–74 30–24 75–79 35–39 80–84 40–44 85–89 45–49 90 or over 50–54 Prefer not to say	**Age** 16–19 55–59 20–24 65–69 25–29 70–74 30–24 75–79 35–39 80–84 40–44 85–89 45–49 90 or over 50–54 Prefer not to say
2021 Census (England & Wales) **What is your sex?** • Female • Male	• ‘Prefer not to say’ added as an option.	**What is the sex you were registered with at birth?** • Female • Male • Prefer not to say	**What is the sex you were registered with at birth?** • Female • Male • Prefer not to say
Question based on LGBT Foundation and Stonewall guidance LGBT Foundation guidance: **Which of the following options best describes how you think of yourself?** • Woman [including trans woman] • Man [including trans man] • Non-binary • In another way The following codes can be used for non-responses: • Not stated (PERSON asked but declined to provide a response) • Not known (not recorded) Stonewall guidance: **Which of the following best describes your gender?** • Female • Male • Prefer not to say • Prefer to self-describe	• ‘Prefer not to say’ and ‘Prefer to self-describe’ added as options	**Which of the following best describes your gender?** • Man • Woman • Non-binary • Prefer to self-describe (specify, if you wish) • Prefer not to say	**Which of the following best describes your gender?** • Man • Woman • Non-binary • Prefer to self-describe (specify, if you wish) • Prefer not to say
2021 Census (England & Wales) **Is the gender you identify with the same as your sex registered at birth?** • Yes • No, write in gender identity	• Question text changed to match Stonewall Guidance.	**Do you identify as trans?** • Yes • No • Prefer not to say	**Do you consider yourself to be trans?** *Trans is a term used to describe people whose gender is not the same as the sex they were registered at birth.* • Yes • No • Prefer not to say
2021 Census (England & Wales) **Which of the following best describes your sexual orientation?** • Straight/Heterosexual • Gay or Lesbian • Bisexual • Other sexual orientation, write in	• Option ‘other sexual orientation, write in’ adapted to ‘Prefer to self-describe (specify, if you wish)’. • ‘Prefer not to say’ added as an option.	**Which of the following best describes your sexual orientation?** • Bisexual • Straight/Heterosexual • Gay or lesbian • Prefer to self-describe (specify, if you wish) • Prefer not to say	**Which of the following best describes your sexual orientation?** *This is not a complete list. Please choose the option that best fits you.* • Bisexual • Straight/Heterosexual • Gay or lesbian • Prefer to self-describe (specify, if you wish) • Prefer not to say
2021 Census (England & Wales) **What is your ethnic group?** White • English, Welsh, Scottish, Northern Irish or British • Irish • Gypsy or Irish Traveller • Roma • Any other White background, write in Mixed or Multiple ethnic groups • White and Black Caribbean • White and Black African • White and Asian • Any other Mixed or Multiple background, write in Asian or Asian British • Indian • Pakistani • Bangladeshi • Chinese • Any other Asian background, write in Black, Black British, Caribbean or African • Caribbean • African background, write in below • Any other Black, Black British or Caribbean background, write in Other ethnic group • Arab • Any other ethnic group, write in	• Options reordered alphabetically. • Option ‘any other background, write in’ adapted to ‘prefer to self-describe (specify, if you wish)’. • ‘Prefer not to say’ added as an option.	**What is your ethnic group?** *Please choose one option that best describes your ethnic group or background* White • English, Welsh, Scottish, Northern Irish or British • Irish • Gypsy or Irish Traveller • Roma • Prefer to self-describe (specify, if you wish) Mixed or Multiple ethnic groups • White and Black Caribbean • White and Black African • White and Asian • Prefer to self-describe (specify, if you wish) Asian or Asian British • Indian • Pakistani • Bangladeshi • Chinese • Prefer to self-describe (specify, if you wish) Black, Black British, Caribbean or African • Caribbean • African background (specify, if you wish) • Prefer to self-describe (specify, if you wish) Other ethnic group • Arab • Prefer to self-describe (specify, if you wish) • Prefer not to say	**What is your ethnic group?** *Please choose one option that best describes your ethnic group or background* Asian or Asian British • Bangladeshi • Chinese • Indian • Pakistani • Prefer to self-describe (specify, if you wish) Black, Black British, Caribbean or African • African background (specify, if you wish) • Caribbean • Prefer to self-describe (specify, if you wish) Mixed or Multiple ethnic groups • White and Asian • White and Black Caribbean • White and Black African • Prefer to self-describe (specify, if you wish) Other ethnic group • Arab • Prefer to self-describe (specify, if you wish) White • English, Welsh, Scottish, Northern Irish or British • Gypsy or Irish Traveller • Irish • Roma • Prefer to self-describe (specify, if you wish) Prefer not to say
2021 Census (England & Wales) **What is your religion?** • No religion • Christian (including Church of England, Catholic, Protestant and all other Christian denominations) • Buddhist • Hindu • Jewish • Muslim • Sikh • Any other religion, write in	• Categories amended from named followers of religion to the religion itself ie Buddhist amended to Buddhism. • ‘Prefer not to say’ added as an option.	**What is your religion?** • No religion • Buddhism • Christianity (including Church of England, Catholic, Protestant and all other Christian denominations) • Hinduism • Islam • Judaism • Sikhism • Any other religion (specify, if you wish) • Prefer not to say	**What is your religion?** • No religion • Buddhism • Christianity (including Church of England, Catholic, Protestant and all other Christian denominations) • Hinduism • Islam • Judaism • Sikhism • Any other religion (specify, if you wish) • Prefer not to say
2021 Census (England & Wales) **What is your main language?** • English • Other, write in (including British Sign Language)	• Question text adapted. • ‘Prefer not to say’ added as an option.	**What is your main language?** • English • Other, (specify, if you wish) • Prefer not to say	**What is your preferred language?** • English • Other, (specify, if you wish) • Prefer not to say
2021 Census (England & Wales) **Census question ‘Have you completed an apprenticeship?’** • Yes • No **Census question ‘Have you achieved a qualification at degree level or above?’** • Yes • No **Census question ‘Have you achieved any other qualifications?’** GCSEs or equivalent • 5 or more GCSEs (A*-C, 9–4), O levels (passes) or CSEs (grade 1) • Any other GCSEs, O levels or CSEs (any grades) or Basic Skills Course AS, A level or equivalent • 2 or more A levels, 4 or more AS levels • 1 A level, 2–3 AS levels • 1 AS level NVQ or equivalent • NVQ level 3, BTEC National, OND or ONC, City and Guilds Advanced Craft • NVQ level 2, BTEC General, City and Guilds Craft • NVQ level 1 Other or no qualifications • Any other qualifications, equivalent unknown • No qualifications	• Question text adapted. • ‘Prefer not to say’ added as an option.	**What is the highest level of education you have completed?** *If you did not complete your education in the UK, please choose the closest equivalent.* • Primary school • Secondary school • College/A-Levels/vocational education • University undergraduate degree/vocational degree • University postgraduate degree • Prefer not to say	**Have you completed any qualifications?** *If you did not complete your education in the UK, please choose the closest equivalent. This is not a complete list. Please choose the options that best fit you.* Please select all that apply: • No formal qualifications • GCSEs or equivalent (e.g., GCSEs, O levels, CSEs, Basic Skills course) • AS, A level or equivalent • NVQ up to level 3 or equivalent (e.g., NVQ level 1–3, BTEC General or National, OND or ONC, City and Guilds Advanced Craft) • Qualification at degree level or above (e.g., university degree, foundation degree, HND, HNC, NVQ level 4 or above, teaching or nursing) • Apprenticeship (e.g., trade, advanced, foundation, modern) • Any other qualifications or equivalent unknown • Prefer not to say
2021 Census (England & Wales) **In the last seven days, were you doing any of the following?** • Working as an employee • Self-employed or freelance • Temporarily away from work ill, on holiday or temporarily laid off • On maternity or paternity leave • Doing any other kind of paid work • None of the above **Which of the following describes what you were doing in the last seven days?** • Retired (whether receiving a pension or not) • Studying • Looking after home or family • Long-term sick or disabled • Other	• ‘Prefer not to say’ added as an option.	**Are you currently:** *Select all that apply* • Employed • Self employed • Not employed and looking for work • Not employed and not looking for work - for example stay at home parent, cannot work, carer. • Retired • Studying • Prefer not to say	**Please select all that apply:** • Employed (including self-employed) • On temporary leave (e.g. parental leave) • Not employed • Studying • Retired • Prefer not to say
Standard Occupational Classification 2010 guidance **Ask respondents to tick one box to show which best describes the sort of work they do. If they are not working now, ask them to tick a box to show what they did in their last job.** • Modern professional occupations such as: teacher – nurse – physiotherapist – social worker – welfare officer – artist – musician – police officer (sergeant or above) – software designer • Clerical and intermediate occupations such as: secretary – personal assistant – clerical worker – office clerk – call centre agent – nursing auxiliary – nursery nurse • Senior managers or administrators (usually responsible for planning, organising and co-ordinating work, and for finance) such as: finance manager – chief executive • Technical and craft occupations such as: motor mechanic – fitter – inspector – plumber – printer – tool maker – electrician – gardener – train driver • Semi-routine manual and service occupations such as: postal worker – machine operative – security guard – caretaker – farm worker – catering assistant – receptionist – sales assistant • Routine manual and service occupations such as: HGV driver – van driver – cleaner – porter – packer – sewing machinist – messenger – labourer – waiter/waitress – bar staff • Middle or junior managers such as: office manager – retail manager – bank manager – restaurant manager – warehouse manager – publican • Traditional professional occupations such as: accountant – solicitor – medical practitioner – scientist – civil/mechanical engineer	• The category options were taken from the Census 2011 derived National Statistics Socio-economic Classification (NS-SEC) and the list was inverted. • ‘Prefer not to say’ added as an option.	**What is your main job, or, if not working, your last main job** • Routine occupation – for example Bar staff, Cleaner, Labourer, Bus driver, Lorry driver • Semi-routine occupation – for example Traffic warden, Receptionist, Shelf-stacker, • Care worker, Telephone salesperson • Lower supervisory and technical occupation – for example Mechanic, Chef, Train driver, Plumber, Electrician • Small employers and own account worker – for example Farmer, Shopkeeper, Taxi driver, Driving instructor, Window cleaner • Intermediate occupation – for example Armed forces up to sergeant, Paramedic, Nursery Nurse, Police up to sergeant, Bank staff • Lower managerial, administrative & professional occupation – for example Social worker, Nurse, Journalist, Retail manager, Teacher • Higher managerial, administrative & professional occupation – for example Lawyer, Architect, Medical doctor, Chief executive, Economist • Never worked and long-term unemployed • Full-time student • Prefer not to say	This question was not included in the final question set.
Daisy questionnaire V2 **Do you consider yourself to be a disabled person?** • Yes • No • Prefer not to say	• Changed ‘be a disabled person’ to ‘have a disability’ based on PPI input within the project team	**Do you consider yourself to have a disability?** • Yes • No • Prefer not to say	**Do you consider yourself to have a disability?** *A disability is a physical or mental condition that has a ‘substantial’ and ‘long-term’ impact on your ability to do normal daily activities.* • Yes • No • Prefer not to say
2021 Census (England & Wales) **Do you have any physical or mental health conditions or illnesses lasting or expected to last 12 months or more?** • Yes • No	• Question text adapted to address patients with cancer. • ‘Don’t know’ added as an option. • ‘Prefer not to say’ added as an option.	**Other than your cancer diagnosis, do you have any physical or mental health conditions or illnesses lasting or expected to last 12 months or more?** • Yes • No • Don’t know • Prefer not to say	**[Other than your cancer diagnosis – PLEASE DELETE IF REQUIRED,] do you have any physical or mental health conditions or illnesses lasting or expected to last 12 months or more?** • Yes • No • Don’t know • Prefer not to say
Department for Work and Pensions Family Resources Survey (FRS) 2021–2022 **Do any of these conditions or illnesses affect you in any of the following areas?** • Vision (for example blindness or partial sight) • Hearing (for example deafness or partial hearing) • Mobility (for example walking short distances or climbing stairs) • Dexterity (for example lifting and carrying objects, using a keyboard) • Learning or understanding or concentrating • Memory • Mental Health • Stamina or breathing or fatigue • Socially or behaviourally (for example associated with autism spectrum disorder (ASD) which includes Asperger’s, or attention deficit hyperactivity disorder (ADHD)) • Other • Refusal (spontaneous) • None of the above (spontaneous)	• ‘Prefer not to say’ added as an option.	**Do any of these conditions or illnesses affect you in any of the following areas?** • Vision (for example blindness or partial sight) • Hearing (for example deafness or partial hearing) • Mobility (for example walking short distances or climbing stairs) • Dexterity (for example lifting and carrying objects, using a keyboard) • Learning or understanding or concentrating • Memory • Mental Health • Stamina or breathing or fatigue • Socially or behaviourally (for example associated with autism spectrum disorder (ASD) which includes Asperger’s, or attention deficit hyperactivity disorder (ADHD)) • Other • None of the above • Prefer not to say	**Do any of these conditions or illnesses affect you in any of the following areas?** • Vision (e.g. blindness or partial sight) • Hearing (e.g. deafness or partial hearing) • Mobility (e.g. walking short distances or climbing stairs) • Dexterity (e.g., lifting and carrying objects, using a keyboard) • Learning or understanding or concentrating • Memory • Mental Health • Stamina or breathing or fatigue • Socially or behaviourally (e.g. understanding social cues or nonverbal skills) • Other • None of the above • Prefer not to say
2021 Census (England & Wales) **On 21 March 2021, what is your legal marital or registered civil partnership status?** • Never married and never registered a civil partnership • Married • In a registered civil partnership • Separated but still legally married • Separated, but still legally in a civil partnership • Divorced • Formerly in a civil partnership which is now legally dissolved • Widowed • Surviving partner from a registered civil partnership	• Categories collapsed to reduce detail. • ‘Prefer not to say’ added as an option.	**What is your current legal marital or registered civil partnership status?** • Never married and never registered a civil partnership • Married or in a registered civil partnership • Separated but still legally married or in a civil partnership • Divorced/formerly in a civil partnership which is now legally dissolved • Widowed • Prefer not to say	This question was not included in the final question set.
Bespoke question	• N/A	**Do you have a caring responsibility either as a parent, grand-parent or legal guardian for a child/children under the age of 16?** • Yes • No • Prefer not to say **If yes, are you currently on parental leave?** • Yes • No • Prefer not to say	**Do you have a caring responsibility for a child/children under the age of 16?** • Yes • No • Prefer not to say
2021 Census (England & Wales) **Do you look after, or give any help or support to, anyone because they have long-term physical or mental health conditions or illnesses, or problems with old age?** • No • Yes, 9 hours a week or less • Yes, 10 to 19 hours a week • Yes, 20 to 34 hours a week • Yes, 35 to 49 hours a week • Yes, 50 or more hours a week	Simplified option categories. ‘Prefer not to say’ added as an option.	**Do you look after, or give any help or support to, anyone because they have long-term physical or mental health conditions or illnesses, or problems related to old age?** • Yes • No • Prefer not to say	**Are you the main carer for anyone because they have long-term physical or mental health conditions or illnesses?** • Yes • No • Prefer not to say

We maintained sex, gender and trans status as three separate questions based on the Stonewall and LGBT Foundation guidance. [27, 28] However, we felt it was important to capture sex at birth as a binary variable as sex has important physiological implications in a clinical setting. Furthermore, people with intersex characteristics are often assigned male or female at birth, making this difficult to capture with existing questions and no comparable national data for intersex frequency was found.

Questions were identified for employment status (England and Wales census), disability (DAISY survey) and education (England and Wales census), but each used multiple items to measure one characteristic. This was considered too long for the purpose of monitoring inclusivity. Therefore, these questions were combined into one to three items each to reduce responder burden.

Socioeconomic position (SEP) and its component characteristics were discussed at length. We considered whether it was possible to collect SEP using combined questions like the NS-SEC. These include free text questions like ‘what is your occupation’, and ‘how many people work in your division.’ However, these require coding expertise which is likely beyond the capacity of most research teams, and evidence suggests that self-report versions have limited reliability [26]. Further, calculation requires responses to numerous questions. The Government Statistical Service recommendations require eight questions related to socio-economic background which we considered to be too extensive to include in our question set [30].

The index of multiple deprivation can be calculated from participants’ postcodes, providing an area-level measure which can be used as a proxy for SEP [31]. Most clinical researchers will have access to postcodes in existing records or collect postcodes along with contact details. Therefore, we decided not to collect self-reported postcode within the demographic question set.

We next considered questions covering component characteristics of SEP, including occupation, employment status, income, and education level. Based on experience in our previous studies and in discussion with our patient and public involvement partners, it was agreed income had poor completeness and was too intrusive. Asking about participants’ parents and family as a young person seemed inappropriate and irrelevant in a clinical trial setting. In contrast, occupation, employment status, and education may directly impact a person’s ability to take part in clinical research based on time availability and understanding of the information. Education has also been used most often as a measure of SEP out of the component characteristics [32]. Therefore, we decided to focus solely on these characteristics.

## Public consultation

In June 2024, the opportunity was advertised through five community/advocacy groups and three patient and public involvement groups. Sixty-two people expressed interest: 51 in the health, disability, and unpaid care group, 48 in the education and employment group, 44 in the ethnicity, language and religion group and 34 in the sexual orientation and gender identity group.

We held the four discussion groups from June to July 2024, with a total of 29 contributors, attending one group each. Numbers were limited by availability, optimum size of discussion groups and funding. Seven of the nine people invited attended the health, disability and unpaid care group, eight of the ten people invited attended the ethnicity, language and religion group, seven of the ten people invited attended the education and employment group and six of the seven people invited attended the sexual orientation and gender identity group. Two quieter contributors from the health, disability and unpaid care group and the education and employment group attended follow-up one-to-one interviews with EL to ensure their input was heard.

Twenty people described themselves in their expressions of interest. At least 10 of these 20 contributors came from a minority ethnic background, 11 had one or more disabilities or long-term health conditions, five were people of faith, three were members of the LGBTQIA+ community, and seven had experiences of unpaid care. Nine people did not provide any personal description.

All discussion groups lasted the full two hours. One-to-one interviews covered all questions in the question set. Changes were made based on the feedback in the project team discussion and the revised version was re-distributed to discussion group contributors. Two people provided additional feedback by email on the revised version.

A summary of the feedback and resulting decisions can be found in Table 4. Overall, three questions were removed: occupation, marital status, and parental leave. Ten questions were modified. The final question set includes 15 questions covering 12 characteristics: age, sex, gender, trans status, sexual orientation, ethnicity, religion, language, education, employment status, disability, long-term conditions and their impact, childcare, and other caring responsibilities.

**Table 4:** Public consultation feedback and resulting decisions made per question

Question for public consultation	Public consultation feedback	Final decision
**Instructions:** No Instructions were given prior to the sessions – we discussed what they should look like throughout the sessions and developed them across the four groups.	Explaining the reason for collection for each of the items was seen as a priority, along with highlighting that all responses are voluntary, and that the data will be kept safe.	The instructions developed throughout the discussion groups were placed before the question set. We considered drafting specific explanatory text for each question, but this was not included due to repetition. We recommend that trial teams draft specific explanations for each question if relevant. Example barriers to participation were initially included, but these caused confusion, so these were removed.
**Age** 16–19 35–39 55–59 75–79 20–24 40–44 60–64 80–84 25–29 45–49 65–69 85–89 30–34 50–54 70–74 90 or over	No concerns with this question.	No changes made.
**2. Sex and gender** What is the sex you were registered with at birth? • Female • Male • Prefer not to say	There were contrasting views about the relevance of this question. Some people felt this was routine, whereas others suggested it might be intrusive.	No changes made.
Which of the following best describes your gender? • Man • Woman • Non-binary • Prefer to self-describe (specify, if you wish) • Prefer not to say	It was suggested to change ‘man’/’ woman’ to ‘male/female’. There was a general agreement to include the non-binary response option and ‘prefer to self-describe’.	We decided to maintain the current wording.
Do you identify as trans? • Yes • No • Prefer not to say	There were mixed views on whether this should be a separate question from gender, though everyone was in agreement that it was important to collect information about being transgender. It was highlighted that the wording is a bit ambiguous – i.e. what does it mean to ‘identify’ as something?	We explored combining the gender and trans questions but it led to a number of complexities. Without doing thorough research, it was unclear how people would understand the new combined question. Therefore, we decided to keep the two separate questions as presented in the census and in line with recommendations from relevant organisations (Table 3). We updated the question wording to match the Scottland 2022 census which did not use the term ‘identify’.
**3. Which of the following best describes your sexual orientation?** • Bisexual • Straight/Heterosexual • Gay or Lesbian • Prefer to self-describe (specify, if you wish) • Prefer not to say	People thought this question might be surprising and that the reason for collection should be explained. People also thought there were some options missing, e.g., sexually fluid, queer, pan sexual, asexual. However, others worried about the cognitive burden of too many options.	We considered adding more options, but on balance this felt overly burdensome and it would still be difficult to be comprehensive. The ‘prefer to self-describe’ option should allow people respond accurately, given the broad range of sexual orientations. We have added brief text to highlight that this list is not exhaustive to avoid the implication that any identities not listed are considered ‘other’.
**4. What is your ethnic group?** *Please choose one option that best describes your ethnic group or background* White • English, Welsh, Scottish, Northern Irish or British • Irish • Gypsy or Irish Traveller • Roma • Prefer to self-describe (specify, if you wish) Mixed or Multiple ethnic groups • White and Black Caribbean • White and Black African • White and Asian • Prefer to self-describe (specify, if you wish) Asian or Asian British • Indian • Pakistani • Bangladeshi • Chinese • Prefer to self-describe (specify, if you wish) Black, Black British, Caribbean or African • Caribbean • African background (specify, if you wish) • Prefer to self-describe (specify, if you wish) Other ethnic group • Arab • Prefer to self-describe (specify, if you wish) Prefer not to say	People were very supportive of the ‘prefer to self-describe’ response options instead of the ‘other’ option within each category. People preferred the response options to appear in alphabetical order. There were some concerns about the inclusion or placement of some specific response options like ‘Roma’ and ‘Arab’. The option to self-describe under the ‘African background (specify, if you wish)’ response option caused confusion.	We ordered the main groups alphabetically.
**5.What is your religion?** • No religion • Buddhism • Christianity (including Church of England, Catholic, Protestant and all other Christian denominations) • Hinduism • Islam • Judaism • Sikhism • Any other religion (specify, if you wish) • Prefer not to say	People thought it was important to collect this data, although there was concern that people might feel targeted therefore it was important to explain why these data were being collected. People wondered why there were only examples for Christianity; it was highlighted that people who are Catholic might not see that as a form of Christianity and that others who practice other religions felt examples weren’t needed. It was also highlighted that some might be missing, e.g., Paganism, Atheism.	We decided to keep the examples for Christianity to match the source question. We also decided not to add further options to avoid making the question set too burdensome. The option to specify should enable capture of any unlisted religions.
**6. What is your main language?** • English • Other (specify, if you wish) • Prefer not to say	There was confusion as to what ‘main language’ meant and how to answer i.e., language spoken at home, mother tongue, or language spoken in everyday life; growing number of people speaking more than one language. The rationale behind this question was unclear. If the purpose was to explore language being a barrier to participation, it might be better to ask this directly.	We changed the wording to ‘preferred’ language. We decided to keep this question despite the complexities of its use due to the importance of language barriers in trial participation.
**7. What is the highest level of education you have completed?** If you did not complete your education in the UK, please choose the closest equivalent. • Primary school • Secondary school • College/A-Levels/vocational education • University undergraduate degree/vocational degree • University postgraduate degree • Prefer not to say	There was a suggestion to include apprenticeships and Scottish equivalent qualifications. Vocational ‘education’ and ‘degrees’ was unclear. There were conflicting views on the relevance of the question, some thought it may seem judgemental and used incorrectly as a as a measure of socioeconomic status while others thought this was directly relevant to literacy levels and the complexity of the trial information. There was concern there was a hidden assumption that people without access to an academic qualification could not take part in trials.	Amended the response options using a combination of the census questions and allowed people to tick all that apply.
**8. Employment** Are you currently: *Select all that apply* • Employed • Self employed • Not employed and looking for work • Not employed and not looking for work – for example stay at home parent, cannot work, carer • Retired • Studying • Prefer not to say	There was concern about the rationale for asking this question and the use of the data. There was confusion as to whether this was a measure of socioeconomic status and whether household income should be asked for instead. However, there were mixed views on how this would be interpreted and whether people would feel comfortable answering such a question.	This question was retained, as we have seen during trial recruitment there is a clear difference between participation by working age. We have amended the options to better reflect the purpose of the question.
**9. Occupation** What is your main job, or, if not working, your last main job: • Routine occupation – for example Bar staff, Cleaner, Labourer, Bus driver, Lorry driver • Semi-routine occupation – for example Traffic warden, Receptionist, Shelf-stacker, Care worker, Telephone salesperson • Lower supervisory and technical occupation – for example Mechanic, Chef, Train driver, Plumber, Electrician • Small employers and own account worker – for example Farmer, Shopkeeper, Taxi driver, Driving instructor, Window cleaner • Intermediate occupation – for example Armed forces up to sergeant, Paramedic, Nursery Nurse, Police up to sergeant, Bank staff • Lower managerial, administrative & professional occupation – for example Social worker, Nurse, Journalist, Retail manager, Teacher • Higher managerial, administrative & professional occupation – for example Lawyer, Architect, Medical doctor, Chief executive, Economist • Never worked and long-term unemployed • Full-time student • Prefer not to say	The general consensus was that this question in addition to the questions about education and employment was too intrusive. The question was felt to be very text-heavy and complicated, and the categories were in a strange order and didn’t match the examples provided. Contributors felt the occupation question was challenging to read and respond to due to the length, complexity of the occupation groupings and difficulty aligning their own work with one of the categories.	This question was removed.
**10. Health** Do you consider yourself to have a disability? • Yes • No • Prefer not to say	People felt this question was important in addition to the question below as it allows people to ‘identify’ as having a disability. There were mixed views on whether the question should include a definition of ‘disability’. There was some confusion about what would be considered a disability i.e. whether a disability would be considered such if symptoms are managed with medication or a device.	An explanation of disability was included, highlighting that it refers to any health conditions which negatively impact daily life. The wording was taken from the Equality Act 2010 as recommended by our patient and public partners. We have not defined whether having medical support for symptoms should change the response as it was considered important for individuals to self-describe.
[Other than your cancer diagnosis – PLEASE DELETE IF NOT REQUIRED] do you have any physical or mental health conditions or illnesses lasting or expected to last 12 months or more? • Yes • No • Don’t know • Prefer not to say	No concerns with this question.	No changes made.
Do any of these conditions or illnesses affect you in any of the following areas? • Vision (for example blindness or partial sight) • Hearing (for example deafness or partial hearing) • Mobility (for example walking short distances or climbing stairs) • Dexterity (for example lifting and carrying objects, using a keyboard) • Learning or understanding or concentrating • Memory • Mental Health • Stamina or breathing or fatigue • Socially or behaviourally (for example associated with autism spectrum disorder (ASD) which includes Asperger’s, or attention deficit hyperactivity disorder (ADHD)) • Other • None of the above • Prefer not to say	We decided to include this question as well as the disability question above, because some people do not identify as disabled but do have a long-term health condition. People were familiar with this list. Contributors suggested adding sensory impairments, allergies and pain. However, it was also felt that extending the list might increase response burden. It was felt the examples for ‘socially or behaviourally’ were offensive because they list conditions rather than impairments and people with those conditions might feel they have to tick that box.	We strongly considered including pain as a response option, however we decided not to as pain is a symptom that might impact people in many of these areas. Adding pain would also complicate mapping responses to existing datasets. We also decided not to add allergies as this is a specific condition rather a way that people can be affected. We have amended the examples for ‘socially or behaviourally’.
**11, Marital status** What is your current legal marital or registered civil partnership status? • Never married and never registered a civil partnership • Married or in a registered civil partnership • Separated but still legally married or in a civil partnership • Divorced/formerly in a civil partnership which is now legally dissolved • Widowed • Prefer not to say	People felt this was an unnecessary and outdated question. However, contributors noted the importance of social support in trial participation. Other, more preferable options could be living status (i.e. alone or not), relationship status or support at home.	This question was removed. We could not find an appropriate question in national surveys to assess social support so no question was added in its place but it was noted that this will be important to consider in future iterations of the question set.
**12. Children and childcare** Do you have a caring responsibility either as a parent, grand-parent or legal guardian for a child/children under the age of 16? • Yes • No • Prefer not to say	Contributors suggested removing ‘parent, grand-parent or legal guardian’ to simplify the question. They also suggested merging this question with the caring responsibilities question below.	We amended the question to remove ‘parent, grand-parent or legal guardian’. The ‘children and childcare’ and ‘caring responsibilities’ sections were placed into the same section but kept as two questions to avoid over-complication.
If yes, are you currently on parental leave? • Yes • No • Prefer not to say	There was confusion about why this question was being asked. It was considered to be more about working status.	Removed this question completely and added parental leave as a response option in the employment status question.
**13. Caring responsibilities** Do you look after, or give any help or support to, anyone because they have long-term physical or mental health conditions or illnesses, or problems related to old age? • Yes • No • Prefer not to say	This question was considered too long and complicated, and the term old age was deemed potentially offensive. It was queried whether this was only relevant if someone is a primary carer rather than capturing if someone provides some/little support.	Simplified the question accordingly.

Three public contributors completed feedback forms. Feedback was generally positive. The contributors felt they were given adequate opportunity to speak and that their contributions made a difference to the project. Suggested improvements included grouping contributors by disease type, sending contributors the presentation slides in advance, and increasing the discussion group length.

## Discussion

Through consultation with diverse patient and public involvement partners and public contributors, we have collated an acceptable set of demographic questions for use in UK-based health research to monitor and assess inclusivity. This helps to fill the gap in national guidance and will provide an important starting point for trialists and researchers in future. Use of these questions will help to standardise and expand the collection of demographic data to ensure we are better able to assess and compare the inclusivity of our research. We plan to evaluate the question set in an ongoing pilot and adapt it in the future as outlined in the ‘next steps’ section below.

Our consultation reaffirmed some general principles of inclusive data collection, including the importance of avoiding the term ‘other’ where possible and providing a ‘prefer not to say’ option for each question [28]. Whilst the latter may lead to missing data, it may help overall response rates if people can choose not to answer certain questions that they find particularly sensitive. The wording changes should not prevent mapping to national datasets. It was also recommended that categories should be alphabetised instead of being ordered by prevalence - the approach taken in the UK Censuses - which could be interpreted as giving more weight or importance to national majorities.

Public contributors consistently emphasised the importance of clearly explaining the reason for collecting each characteristic, and that all answers will remain confidential. Providing a strong justification may make respondents feel more comfortable offering personal information. Throughout the project, we iteratively developed introductory text to explain the overall purpose of data collection to monitor inclusivity. However, we recognise that the purpose and importance of each characteristic will differ depending on the context of the specific research. As this project reviewed the questions outside of the context of a specific trial, our attempts at developing wording to justify the collection of each question became repetitive. We recommend that researchers using the question set consider adapting the introductory text and/or adding a brief and simple justification for collecting each question in collaboration with PPI advisors on their specific projects.

The question set does not capture all protected characteristics or potentially underserved groups suggested by INCLUDE, however, this list balances responder burden while capturing characteristics that may relate to inclusion in health research. Researchers should consider whether there are other important underserved groups who may be excluded from their specific projects, and whether additional demographic items may be needed alongside this question set.

While contributors suggested marital status was too intrusive and irrelevant for collection in a research context, they did think that related concepts like social support could be important in understanding inclusion. We were unable to find a useful question for social support with comparable national data, but this should be considered for future additions to the question set.

The best measurement of SEP is widely debated by researchers, and each has their own advantages and limitations [33]. Our question set currently only captures a small number of single-item measures of SEP (e.g. education level). This is because the number and type of questions required to calculate a composite score (i.e. combining a variety of indicators to produce an overall result) were too numerous, irrelevant, or intrusive for the purpose of this question set. Contributors were also strongly against collecting occupation because of the complexity and challenges of self-categorising their occupation into the response options. However, the use of composite scores may be preferred where SEP is to be used in outcome analysis, which researchers could use in place of the education and employment questions currently included in the question set. Alternatively, they may wish to collect additional SEP data alongside the question set items where this is most appropriate.

Whilst evidence suggests that free text is preferable to the public for collecting ethnicity and some other demographic data, challenges still exist in collecting, coding, and analysing this data [34]. We recognise the importance of self-expression and acknowledge the restricted nature of categorical response options. Indeed, public contributors in our focus groups found that some ethnicity categories were missing or were too specific. However, we also recognise the need for a standard solution in research today. As new evidence emerges, we plan to revise this question set. For example, the census categories for ethnicity are currently undergoing review by the Government Statistical Service Harmonisation Team and research is ongoing in the use of artificial intelligence to code and interpret free text response data. We hope that these improvements can be incorporated into our question set in the near future.

Data collection alone is not sufficient to address inequity of access and representation in health research. However, by routinely collecting these data, we should be able to identify which groups are currently missing from our studies. This will help to provide a benchmark against which we can improve inclusion in the future. Monitoring these data during recruitment may also allow researchers to intervene early, targeting resources to underserved groups and actively improve inclusivity. Expanded data collection across demographic variables will also allow us to consider intersectionality when thinking about inclusivity.

UK regulators, funders, and journals are increasingly emphasising the importance of inclusivity. They recommend the collection and publication of demographic data, which is currently underreported [18, 35–37]. Without a consistent approach, it will become challenging to manage, identify patterns, and measure the impact of any interventions to improve inclusivity. It is also important to recognise that whilst publication of disaggregated data is welcome to aid transparency and accessibility for future meta-analyses, any comparative analysis of outcomes by demographic characteristic is likely to be substantially underpowered for any given study, particularly for groups which represent very small subsets of the UK population.

Our long-term aim is to develop a core demographic question set for use in UK research. Our consultation explored public views on the types of questions that are suitable for demographic data collection, but to be confident in the questions we need further testing and evaluation in a variety of research settings. Our next step will be to share the question set with research teams across the UK and collate feedback and data on its performance to further revise the questions where necessary. Our plans for evaluating the question set are outlined in the ‘next steps’ section below.

## Strengths and limitations

A key strength of our consultation is that it is very timely. Trial Forge (the “PRO EDI” initiative) has recently released guidance on what demographic information should be considered in systematic reviews [14, 38]. The reporting of six core characteristics (age, sex, gender, race, ethnicity, and ancestry, socioeconomic status, and geographic location) has been recommended, to give greater transparency of research populations and their representativeness of the disease being studied. These categories align with the data collected within the DISTINCT question-set, despite being developed independently.

Patient and public involvement was integral to this project. Our two patient and public involvement partners were active members of the project team and contributed to the initial selection of items, the development of recruitment materials and discussion guides, they co-facilitated the discussion groups, revised discussion group materials between sessions and helped make decisions based on discussion group feedback. We also recognised the need for a wide range of public views on the questions, given the focus of the work. Therefore, we expanded our outreach to different communities by advertising across institutions and through relevant specialist advocacy groups. The information provided by our contributors suggests this approach was broadly successful. However, we recognise that many participants may have had or been affected by cancer as our recruitment materials were designed with a focus on cancer research.

We also recognise that there is room for further feedback from other under-served groups as our consultation was limited in size. Further consultation with people from LGBTQ+ backgrounds and those experiencing language, literacy or digital barriers would be beneficial. We did involve people with lived experience of disability in the project but there is likely to be more work needed to refine these questions due to the range of health conditions they cover. Going forward we will also consider alternative formats of the question set to make it more accessible to certain groups (i.e. braille or larger font sizes for the visually impaired).

The childcare responsibility question is bespoke as we were unable to identify an appropriate item from UK surveys. This means there is little data showing that the question is well-understood by respondents and national data may not be directly comparable.

The two teams leading this work are focused on cancer or cancer screening research among adults. Therefore, the question set is likely most relevant in this context and may require adaptation for use in other disease areas. The question set has also been designed specifically for use within the UK and is not applicable to research abroad. We also recognise that other countries may have different approaches to demographic data collection and may or may not approve of the collection of this personal information.

The approach described here provides a structured and replicable methodology for adapting demographic items to new populations. Rapid methods suit the practical nature of this project where we needed to gather and act on feedback iteratively and work collaboratively. However, any modification inherently comes with risks as respondents may interpret questions or response options differently. As such, the final questions presented here need further evaluation which we discuss below.

## Next steps

The question set is now available for use in non-commercial UK health research, alongside guidance for study teams on how to implement the questions in their research, based on recommendations of our PPI contributors (to access the question set contact: distinct@icr.ac.uk).

We plan to evaluate and improve the question set by gathering feedback from research teams using the questions in this early phase of implementation. This will include capturing further views from patient and public involvement partners and members of the public for specific studies, changes study teams make to recommended wording and addition/removal of questions to adapt the survey for specific disease areas. We plan to also examine completeness of each item in prospective research, conduct further cognitive debriefing interviews, and incorporate updated recommendations from relevant national organisations (e.g. the Office for National Statistics) to inform future iterations of the question set.

Using this question set in studies and trials across the UK could help to improve standardisation, allowing for wider monitoring of inclusivity of UK health research. While it may be important to modify, add or remove questions based on investigator and PPI input, retaining the questions as closely as possible to how they are presented should ensure comparability with national data sets and other research using this list. Should uptake of the pilot be widespread, we hope that feedback from other groups will facilitate further refinement, working with patients and the public, the academic trials community, funders, oversight authorities, and publishers to reach consensus on a minimum standard for collection and monitoring in UK research.

## Electronic supplementary material

Below is the link to the electronic supplementary material.

Supplementary material 1

## Data Availability

The DISTINCT Question Set is available on request to distinct@icr.ac.uk. Master RAP sheets are also available on request.

## Abbreviation

CPTU: Cancer Prevention Trials Unit (at QMUL)
ICR-CTSU: The Clinical Trials and Statistics Unit at the Institute of Cancer Research
LGBT: Lesbian, Gay, Bisexual, Transgender
LGBTQIA+: Lesbian, Gay, Bisexual, Transgender, Queer, Intersex, Asexual
NS-SEC: National Statistics Socio-Economic Classification
PPI: Patient and public involvement
QMUL: Queen Mary University of London
RAP: Rapid assessment process
SEP: Socioeconomic position
