# Supplementary material for: Establishing a set of acceptable demographic questions for use in health research through public consultation
Source: Res Involv Engagem. 2026 Jan 28;12:24. doi: 10.1186/s40900-026-00836-1 (PMC12924441; doi:10.1186/s40900-026-00836-1)
Supplement: Supplementary file 1 — Supplementary material 1 [file 40900_2026_836_MOESM1_ESM.docx]

**SUPPLEMENTARY MATERIAL**

**Appendix 1**: Patient and public contributor recruitment materials

**1a**. Infographic

 
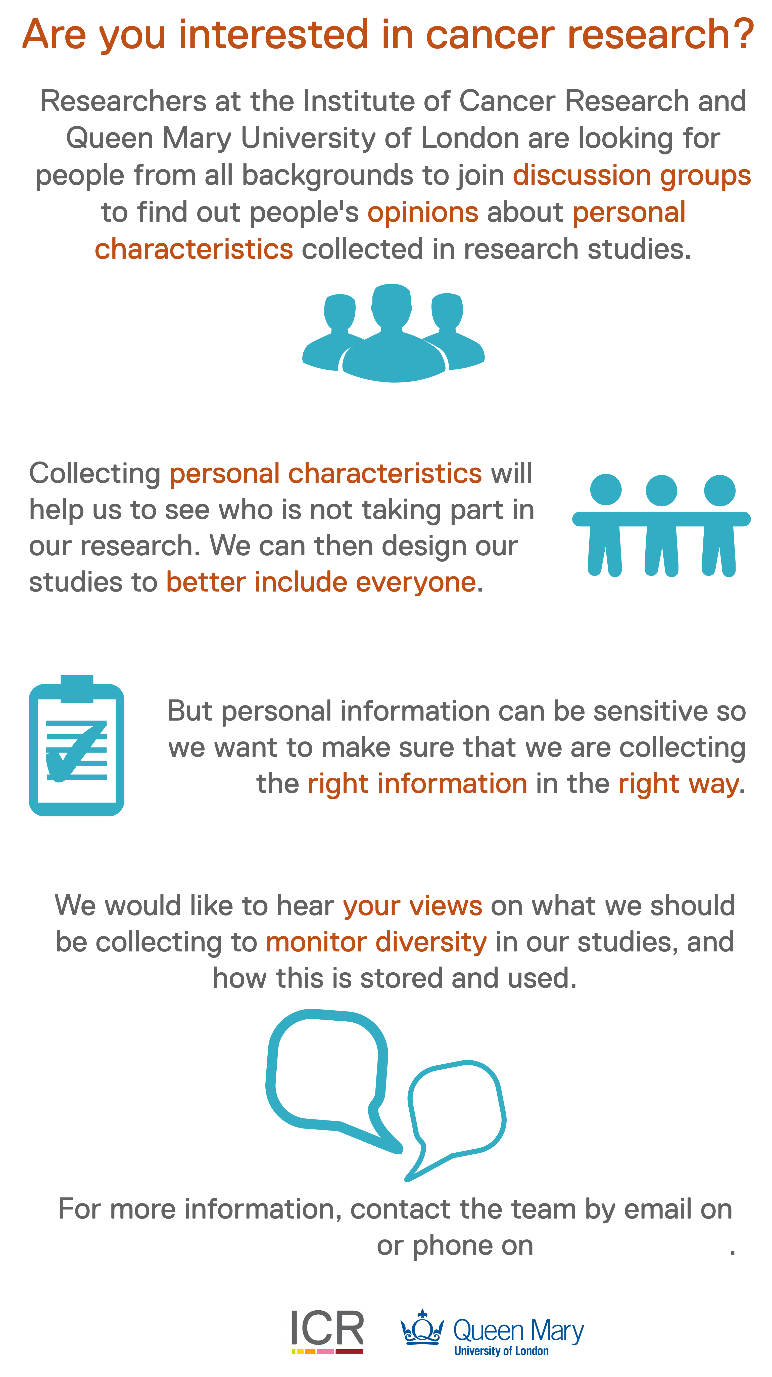


**1b.** Flyer


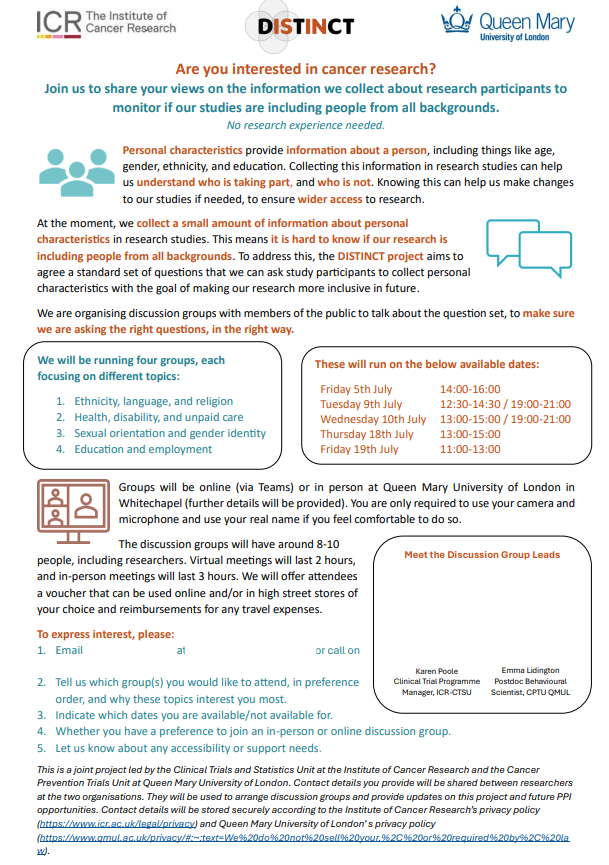


**Appendix 2**: Rapid Assessment Process (RAP) sheet template


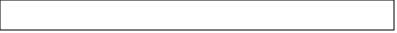
Researcher name


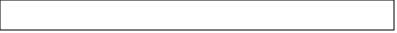
Discussion group

| Initial thoughts |  |
| --- | --- |
| Explanation for collection |  |
| Age |  |
| Sex and gender |  |
| Sexual orientation |  |
| Ethnicity |  |
| Religion |  |
| Main language |  |
| Education |  |
| Employment status |  |
| Occupation |  |
| Health |  |
| Marital status |  |
| Children & childcare |  |
| Caring responsibilities |  |
| Overall thoughts |  |
| Questionnaire instructions |  |
| Data storage |  |
